# Translation and psychometric validation of the Heart Failure Symptom Tracker (HFaST)

**DOI:** 10.1186/s12872-024-04424-7

**Published:** 2024-12-23

**Authors:** Maria Inês Perez, Joana Seringa, Teresa Magalhães

**Affiliations:** 1 NOVA National School of Public Health, NOVA University Lisboa, Lisbon, Portugal; 2 NOVA National School of Public Health, Public Health Research Centre, Comprehensive Health Research Center, CHRC, REAL, CCAL, NOVA University Lisboa, Lisbon, Portugal; 3 Escola Nacional de Saúde Pública, Universidade Nova Lisboa, Avenida Padre Cruz, Lisbon, 1600-560 Portugal; 4 Physical Medicine and Rehabilitation Service, Hospital de Santa Marta, Unidade Local de Saúde São José, Lisbon, Portugal

**Keywords:** Heart failure, Symptom tracker, Disease management, Psychometric validation

## Abstract

**Background:**

Heart Failure (HF) is a global public health issue with high morbidity and mortality rates. Symptom management improves HF patients’ quality of life and demonstrates a potential reduction in hospitalisation, particularly among individuals aged 65 and over. Early identification of patients at higher risk of hospitalisation is essential to guide patient-centred interventions. This study aimed to translate, cross-culturally adapt and evaluate the psychometric properties of the Heart Failure Symptom Tracker (HFaST) tool for the Portuguese population. Additionally, it aimed to test the hypothesis that higher scores of the HFaST are associated with increased hospitalisations due to HF decompensation.

**Methods:**

This cross-sectional study was conducted in two phases. The first phase involved the linguistic translation and cross-cultural adaptation of the HFaST tool into European Portuguese. Content validity was assessed by a panel of ten experts, who evaluated the clarity, relevance and equivalence of the pre-final version. A pre-test, using cognitive interviews with a sample of forty individuals was conducted to assess the item comprehensibility of the adapted tool. The second phase involved the psychometric validity in a sample of sixty HF patients. Participants completed a demographical and clinical assessment, the Portuguese version of the HFaST tool and the Portuguese version of the KCCQ-23 questionnaire. Additionally, the association between HFaST scores and HF hospitalisations were analysed.

**Results:**

Equivalence between versions showed substantial to perfect agreement, with *Fleiss’ k* ranging from 0.678 to 1.000. Necessary adjustments were performed. Pre-test confirmed 95% comprehensibility. Internal consistency was acceptable, with a Cronbach’s Alpha of 0.724, moderate to strong inter-item correlations, and significant correlations between the HFaST and the KCCQ-23 items were observed. Higher HFaST scores were significantly associated with increased hospitalisations, highlighting its role as a predictive tool for clinical risk stratification.

**Conclusions:**

The Portuguese version of the HFaST demonstrated to be a reliable and valid self-management tool for HF patients in Portugal. By predicting the likelihood of hospitalisation risk, the HFaST enables clinicians to implement early interventions, potentially reducing hospital admissions, improving patients’ outcomes and contributing to a better quality of life.

**Clinical trial number:**

not applicable.

## Background

Heart Failure (HF) is a well-acknowledged global public health problem with high prevalence, affecting over 64 million people worldwide and revealing repercussions in terms of high hospital admission rates, with nearly 50% facing readmissions in the first year after diagnosis, imposing high costs for healthcare systems and a negative impact on HF patients’ quality of life (QoL), especially those aged 65 and over [1–4].

It is estimated that approximately 4.4% of the Portuguese population has HF, and recent studies have anticipated an increase of 28% in the burden of HF and 73% in mortality in mainland Portugal by 2036 5–7.

Fatigue, shortness of breath, swelling and reduced tolerance to physical activity are the main cardinal symptoms of HF [1, 2].

According to recent clinical guidelines of the European Society of Cardiology, self-management strategies are considered Class I, Level A evidence-based recommendations for reducing the risk of HF hospitalisation and mortality [1].

Effectiveness in symptom management and monitoring is crucial for better QoL outcomes in HF patients, and several studies highlighted the importance of focusing on symptom recognition, empowerment and patients’ education to improve self-care and prevent or reduce unnecessary hospitalisations [8–12].

On the other hand, the prompt progress in digital health technologies and the relevant position that mobile health (mHealth) has taken on modern medicine might improve self-monitoring, allowing better healthcare outcomes in disease management and the possibility of sharing data with health professionals [12–16].

Current evidence strongly supports the use of the Kansas City Cardiomyopathy Questionnaire (KCCQ) and the Minnesota Living with Heart Failure Questionnaire (MLHF) as the most suitable disease-specific patient-reported outcome measures (PROMs), because of their proven reliability and effectiveness in capturing key aspects of HF signs and symptoms [17].

However, these PROMs often involve lengthy questionnaires in multiple domains and numerous items to answer. They typically include extended recall periods of over seven days [18]. Moreover, while these questionnaires provide insights into symptom recognition and patients’ QoL, they remain limited by not prioritising predictive outcomes such as hospitalisations, which are critical for enhancing HF management strategies.

Several symptom-tracking tools have been developed to meet the increasing demand for user-friendly and effective symptom management in HF patients, without imposing a substantial burden on the user. Among these is the American Heart Association (AHA) tool, designed to prompt immediate action based on symptom severity [19].

The Heart Failure Symptom Tracker (HFaST), was chosen for its research-oriented design and comprehensive assessment capabilities and emerged as a solution to this challenge as a concise, self-administered 6-item tool that effectively allows patients to manage their HF symptoms. It not only facilitates self-care practices but also helps identify individuals at higher risk of hospitalisation, aligning with the main goal of preventing unnecessary hospitalisations [1, 8, 20].

The present study was conceived by considering the high potential and benefits of this instrument, making it an ideal choice for both clinical practice and research, allowing better self-management and the possibility of integrating this tool as part of a model for predicting the risk of hospital admissions, thus addressing a critical unmet need in HF care.

This study aimed to translate, cross-culturally adapt and evaluate the psychometric properties of the HFaST tool for the Portuguese population, allowing this tool to be used for symptom self-management and efficient monitoring by individuals diagnosed with HF in Portugal. The second aim was to analyse whether higher scores on the Portuguese version of the HFaST tool are associated with hospitalisation risk due to HF decompensation.

## Methods

### Design overview

**Fig. 1:**
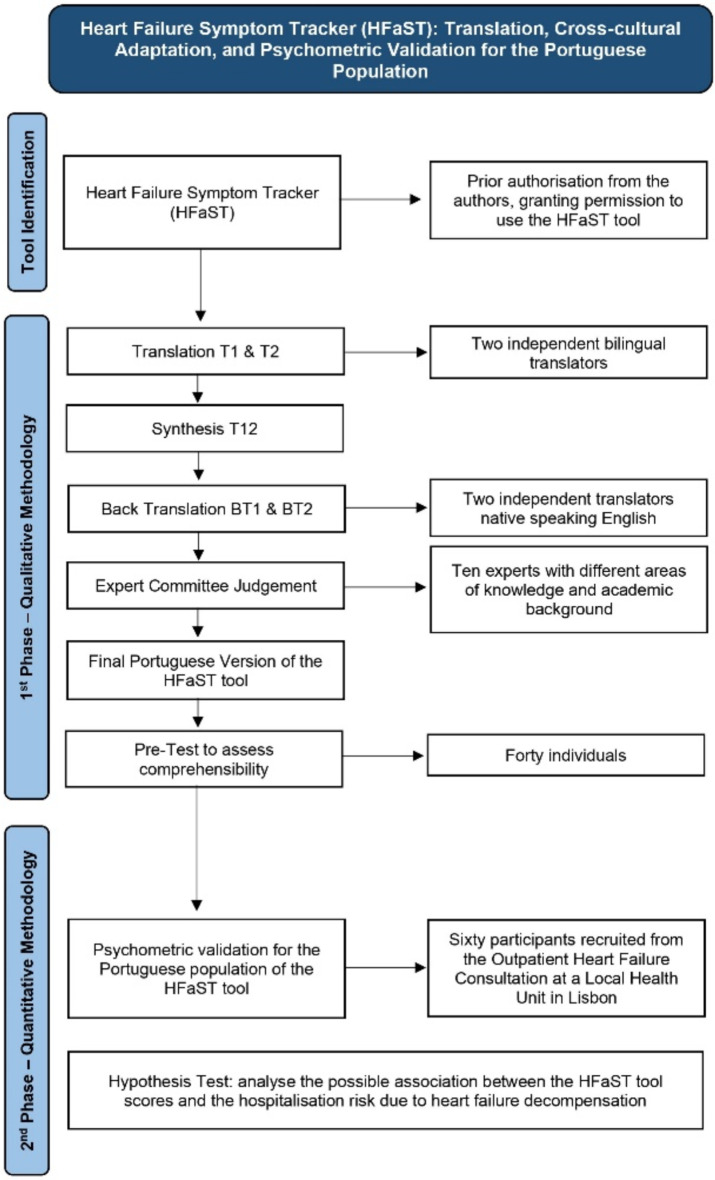
Flowchart for this study

This observational, cross-sectional descriptive study was divided into two phases: first, following a qualitative methodology, the linguistic process of translation into European Portuguese and cross-cultural adaptation of the HFaST tool and, second, following a quantitative methodology, the psychometric validation of the HFaST tool for the Portuguese population. Moreover, this study analysed the possible association between the HFaST tool scores and HF hospitalisations through a quantitative methodology. A flowchart of this study is presented in Fig. 1.

This study had prior authorisation from the authors, granting permission to use the HFaST tool for this specific purpose.

### Qualitative methodology

#### Translation and cross-cultural adaptation of the HFaST tool

The linguistic process of translation and cross-cultural adaptation of the HFaST tool for European Portuguese followed the guidelines proposed by Beaton D. et al. [21], consisting of the following stages:

**Stage I** – translation T1 and T2 were carried out in the target language, independently by two of the authors of this study, both with clinical knowledge but different backgrounds.

**Stage II** – comparison of both translated versions resulted in a synthesised version T12.

**Stage III** – back-translation BT1 and BT2 were carried out by two independent native English speakers without medical background or prior knowledge of the study’s goals.

**Stage IV** – review of all versions by a group of expert judges, which included a team of multidisciplinary professionals, to evaluate the clarity, relevance and equivalence of the instruction, items and response format, guarantying the suitability of the translation and ensuring the content validity of the instrument across different cultures, leading to the development of the final version of the instrument in the target language [21–24].

**Stage V** – pre-test using cognitive interviews in a convenience sample of ordinary individuals with different ages, levels of education and residences across Portugal to evaluate comprehensibility and general acceptance of the scale items.

**Stage VI** – submission of an executive summary with the main results of the European Portuguese translation and cross-cultural adaptation of the HFaST tool to the authors.

#### Procedure

Experts were invited by e-mail to assess the clarity and relevance of each survey item using a five-point Likert scale. The e-mail also included an overview of the study and the informed consent form. The survey had fifteen questions, and qualitative observations from experts were also considered. The experts were provided with a one-month timeframe and two follow-up reminders were sent, one and three weeks after the start of the survey.

After the expert committee’s assessment and considering the qualitative observations on clarity and relevance, adjustments deemed necessary were made, and a pre-test of the Portuguese version of the HFaST was carried out, using cognitive interviews with dichotomous yes/no responses on a non-probabilistic convenience sample of forty individuals of varying ages, levels of education, different professional backgrounds and various geographical areas across Portugal to assess the comprehensibility of each item.

### Quantitative methodology

#### Psychometric validation of the HFaST tool for the Portuguese population

To evaluate the psychometric properties, data was collected from the Outpatient Heart Failure Consultation at a Local Health Unit in Lisbon, Portugal, between March and May 2024. Patients were invited to participate if they met the following inclusion criteria: individuals of both genders, over eighteen years old, diagnosed with HF and followed up at the outpatient heart failure consultation. Patients were excluded if they could not read, answer or complete simple questionnaires.

To ensure data availability for scale validation, the sample size was calculated based on the recommendation of ten respondents per survey item [23, 25].

Each participant received a document containing information about the research project and the informed consent declaration to participate in this study voluntarily. They were also informed that the data collected from their electronic medical records would only be used for this study.

#### Association between the HFaST tool scores and HF hospitalisations

Decompensated HF patients have a higher symptom burden, an increased risk of subsequent mortality, more frequent hospital readmissions and poor quality of life [26].

Johansson I. et al. highlighted in their study that assessing health-related QoL with the Kansas City Cardiomyopathy Questionnaire was a robust predictor of hospital readmission in HF patients, with lower scores linked to a higher risk of negative outcomes [27].

To understand whether there was an association between the score on the symptom assessment tool under study and hospitalisations, we hypothesised that higher scores on the Portuguese version of the HFaST tool, which indicates the presence of more severe symptoms, are associated with a greater hospitalisation risk due to HF decompensation. To analyse this association, data was collected retrospectively from the electronic medical records, covering the period between March 2023 and March 2024.

By analysing this, we hope to ensure that the scale under study can be applied in an early intervention context, avoiding unnecessary hospitalisations.

### Instruments

The following measures were used in this study and each participant was asked to answer each questionnaire by selecting only one option per response item:

#### Baseline demographic and clinical assessment of the study participants

A baseline evaluation was conducted through a questionnaire administered to all participants who agreed to join the study. This questionnaire collected sociodemographic information, including age, gender, marital status, and academic qualifications.

Additionally, clinical data, including the aetiology of HF, the New York Heart Association (NYHA) classification, left ventricular ejection fraction (LVEF), and history of HF hospital admissions in the past year, was collected from electronic medical records. Among these variables, most were used uniquely to characterise the sample, except for the history of HF hospital admissions, which was used to correlate with HFaST scores.

### The Portuguese version of the HFaST tool

The translated and culturally adapted version of the HFaST was administrated to all participants. This self-administered 6-item questionnaire covers concepts of fatigue, shortness of breath, swelling and rapid weight gain and uses a 6-point Likert scale, except for the last item, which involves a dichotomous yes/no answer.

The scale score ranges from 1 to 27, with a high score representing a worse health status, more severe symptoms and the need for clinical care advice.

#### The Portuguese version of the Kansas City cardiomyopathy questionnaire (KCCQ-23)

The Portuguese version of the KCCQ-23 was applied to all the participants. This 23-item questionnaire, a golden standard measure covering five domains, aims to characterise health-related QoL in HF patients. It enables concurrent validation by correlating KCCQ-23 items with HFaST items, following the original HFaST psychometric evaluation methodology [20, 28].

The KCCQ-23 score ranges from 0 to 100, with higher scores indicating better health-related QoL: 0 to 24 indicate very poor to poor, 25 to 49 reflect poor to fair, 50 to 74 signify fair to good, and 75 to 100 represent good to excellent [29–31].

### Patient and public involvement

Patients or the public were not involved in this research’s design, conduct, reporting, or dissemination plans.

### Statistical analysis

To assess content validity, the content validity index (I-CVI and S-CVI) and Fleiss’ k coefficient were performed to provide the degree of agreement between experts. An I-CVI ≥ 0.78 is acceptable for evaluating individual items, and a S-CVI for overall evaluation must have a minimum agreement of 0.80 [32]. The interpretation of Fleiss’ k values followed the categorisation by Landis & Koch [33].

Descriptive statistics were used to describe and characterise the sample under study and to evaluate pre-test data, using frequency, central tendency, dispersion, and maximum and minimum measures.

Cronbach’s Alpha (α) was calculated to assess internal consistency. An α ≥ 0.8 is considered satisfactory; nevertheless, for scales developed in the last five years or with a small number of items, an α ≥ 0.6 is acceptable [23, 34].

Inter-item correlations were used to assess item homogeneity. Coefficients between 0.30 and 0.70 were considered acceptable [35].

The Shapiro-Wilk normality test was conducted to assess the normality of the data distribution.

Spearman’s correlation was used to assess concurrent validity to estimate an association between the Portuguese version of the HFaST and the Portuguese version of the KCCQ-23, a *gold-standard* questionnaire [25]. The correlation coefficient indicates both the direction and strength of a relationship between variables. The sign shows a positive or negative direction, while the value, ranging from − 1 to + 1, denotes the strength [36]. 

The Mann-Whitney Test was used to test the hypothesis of the relationship between hospitalisations due to HF decompensation and the score obtained on the Portuguese version of the HFaST tool.

Data analyses were performed using IBM SPSS Statistics for Windows Version 29.0 and statistical significance was defined as a *p*-value *<* 0.05, with 95% confidence.

## Results

### Characteristics of the expert judges and participants

A non-probabilistic convenience sampling method was used to recruit ten experts comprising different areas of knowledge (four medical doctors, three nurses, one social work assistant, one project manager and one pharmacist) and academic background (30% reported having a postgraduate specialisation, 40% a master degree and 30% a doctoral degree), with a mean age of 44.5 ± 7.96 years and a mean work experience of 18.8 ± 7.45 years, to provide their opinions and make their judgement regarding the content validity of the HFaST tool for the Portuguese population.

For the psychometric validation, this study included a total of sixty participants, comprising 60% males and 40% females, with a mean age of 63.75 ± 11.77 years. Regarding marital status, 61.7% were married or in a common-law marriage. Concerning educational attainment, 35% of participants had attended high school.

The predominant aetiology of HF among the participants was ischaemic, corresponding to 43.3%. Most participants were classified as NYHA Class I (36.7%) and Class II (50%). The LVEF distribution among participants revealed that 66.6% exhibited Reduced Ejection Fraction (HFrEF), defined as LVEF ≤ 40%. In contrast, 16.7% had Preserved Ejection Fraction (HFpEF), defined as LVEF ≥ 50%.

23.4% of participants reported a history of HF hospital admission within the last year. Among these participants, the number of hospital admissions ranged from 1 to 3.

The demographic and clinical characteristics of the study population are presented in Table 1.

**Table 1:** Participants’ baseline characteristics

Participants Characteristics	*N* (%)
**Participants’ Age (in years)**	
(*n* = 60), M*ean (± SD)*	63.75 ± 11.77
**Gender**	
Female Male	24 (40) 36 (60)
**Marital Status**	
Married/Common-law marriage Single Divorced Widowed	37 (61.7) 8 (13.3) 10 (16.7) 5 (8.3)
**Academic Qualifications**	
Primary School Middle School High School Higher Education	16 (26.7) 9 (15.0) 21 (35.0) 14 (23.3)
**Aetiology of HF**	
Ischaemic Valvular Hypertensive Congenital Heart Disease Arrhythmic Other	26 (43.3) 7 (11.7) 5 (8.3) 3 (5.0) 3 (5.0) 16 (26.7)
**NYHA Classification**	
Class I Class II Class III	22 (36.7) 30 (50.0) 8 (13.3)
**Left Ventricular Ejection Fraction**	
≤ 40% 41–49% ≥ 50%	40 (66.6) 10 (16.7) 10 (16.7)
**History of hospital admission in the last year**	
Yes No	14 (23.3) 46 (76.7)
**Number of hospital admissions within the last year (March 2023 - March 2024)**	
0 1 2 3	46 (76.7) 8 (13.3) 5 (8.3) 1 (1.7)

### Translation, content validity through expert judgement and Pre-test

During the translation process, only one major discrepancy arose with two terms presented in the *Likert* scale options: T1 translated “somewhat” as “*um tanto*” and “slightly” as “*levemente*”, while T2 translated as “*um pouco*” and “*ligeiramente*”. The researchers’ team concluded that “*um pouco*” and “*ligeiramente*”, were the best translations for “somewhat” and “slightly”, respectively, in the synthesised version T12. Regarding the back-translation process, no notable inconsistencies were observed between the two back-translations conducted based on the T12 synthesis version. Furthermore, BT1 and BT2 demonstrated a high approximation to the original version of the HFaST tool.

An I-CVI of 0.70 and 1.00 were obtained for the clarity and relevance of the instructions for filling in the Portuguese version of the HFaST tool, respectively. The experts, in their qualitative observations, emphasised the need to shorten the instructions and adjustments deemed necessary were made to ensure better understanding and clarity.

Regarding the clarity and relevance of the *Likert* scale used in the tool, a minimum I-CVI of 0.5 and a maximum of 0.9 were obtained. *“Somewhat better than usual/Um pouco melhor do que o habitual”* and *“Somewhat worse than usual/Um pouco pior do que o habitual”* were the two items with the lowest I-CVI and the experts’ panel suggested eliminating both items, expressing that it could lead to patient confusion and a dubious choice.

*Fleiss’ k* values for clarity and relevance of the *Likert* scale revealed a moderate strength of agreement, 0,548 and 0,570, respectively, supporting the decision to eliminate these two items from the *Likert* scale, leaving it with six options to choose from.

Experts expressed their opinion on the equivalence of the items between the original version and the translated version, revealing a strength of agreement between substantial and perfect, as shown in Table 2.

**Table 2:** Strength of agreement among experts for each item of the Portuguese Version of the HFaST tool, for a *p* < 0.001

HFaST Items	Fleiss’ k	Strength of Agreement (Landis & Koch, 1977)
**HFaST Item 1** – Fatigue or low energy level when performing everyday activities (Fadiga ou pouca energia ao realizar atividades diárias)	0,678	Substantial
**HFaST Item 2** – Shortness of breath when performing everyday activities (Falta de ar quando realizar as atividades diárias)	0,792	Substantial
**HFaST Item 3** – Shortness of breath at rest (Falta de ar em repouso)	0,857	Almost Perfect
**HFaST Item 4** – Shortness of breath while lying down or reclining (for example, needing to add pillows or move to a recliner to sleep) (Falta de ar quando deitado ou reclinado (por exemplo, necessidade de adicionar almofadas ou de se mudar para um cadeirão reclinável para dormir)	0,898	Almost Perfect
**HFaST Item 5** – Swelling of feet, ankles, legs, or abdomen; shoes or waistband feeling tight (Inchaço dos pés, tornozelos, pernas ou abdómen; sensação de sapatos ou cinto apertados)	0,839	Almost Perfect
**HFaST Item 6** – Have you gained more than 2 pounds during the past 24 h or more than 5 pounds during the past 72 h? (Ganhou mais de 1 kg durante as últimas 24 horas ou mais de 2 kg durante as últimas 72 horas? )	1,000	Perfect

**Fig. 2:**
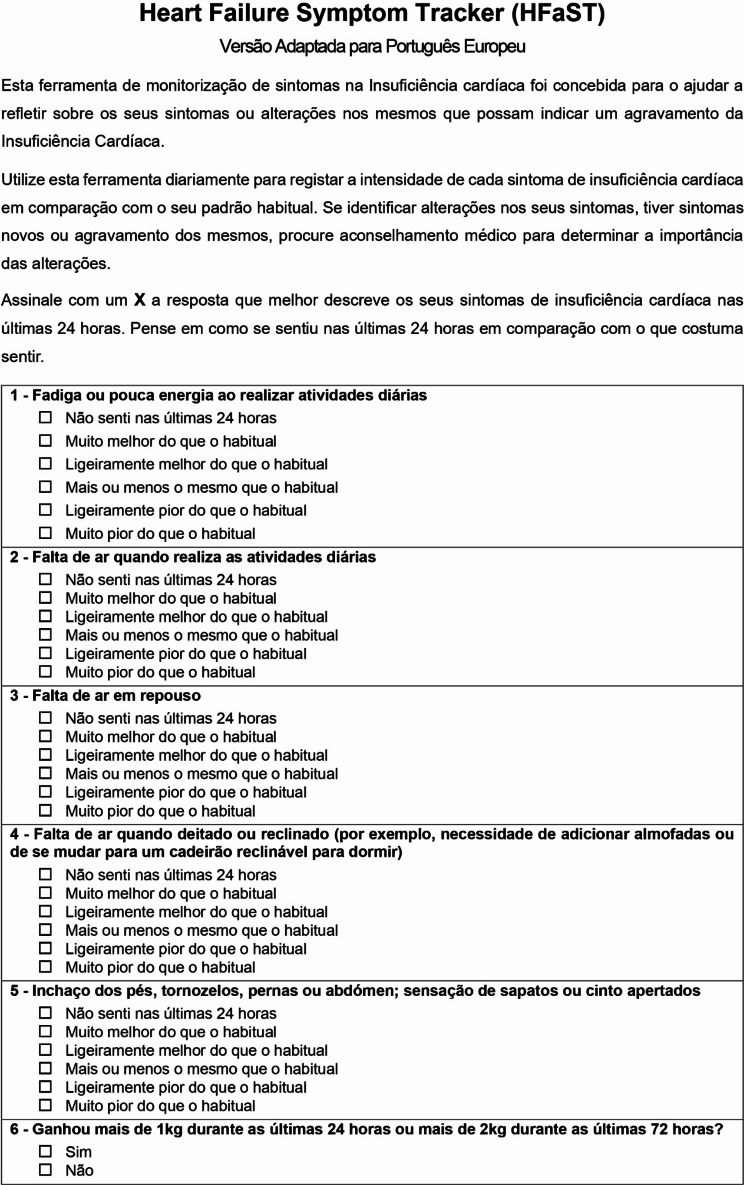
The final Portuguese version of the HFaST tool

In addition, an overall scale content validity index, S-CVI of 0,88, was assessed. These results, along with experts’ qualitative assessments and recommendations, contributed to the ultimate development of the Portuguese version of the HFaST, presented in Fig. 2.

The final instrument was administered through a short cognitive interview, applying a verbal probing technique with dichotomic yes/no answers, to forty individuals to determine the degree of understandability and comprehensibility of the adapted instrument to European Portuguese. The final Portuguese version of the HFaST tool achieved high results, with 95% overall comprehensibility and 100% ease of completion form.

### Psychometric properties assessment

#### Item responsiveness

The distribution of the HFaST response patterns for each item, is detailed in Table 3. The acceptability of the HFaST tool was 100%, with all participants responding to the questionnaire items. For Item 1 – Fatigue or low energy level when performing everyday activities, responses predominantly clustered around scores 2 and 3 (60%), suggesting that fatigue was slightly better or about the same as usual for most of the participants. Notably, 16.7% reported a slight worsening of fatigue compared to their usual state. For Item 2 – Shortness of breath when performing everyday activities and Item 3 – Shortness of breath at rest, 51.7% and 81.7% of participants, respectively, did not report experiencing shortness of breath during activity or at rest in the past 24 h. In Item 4 – Shortness of breath while lying down or reclining, although 55% of participants indicated they did not experience shortness of breath while lying down or reclining in the past 24 h, 25% reported that their orthopnoea was approximately the same as usual. In Item 5 – Swelling of feet, ankles, legs, or abdomen; shoes or waistband feeling tight, responses showed that 70% of participants did not exhibit swelling in the feet, ankles, legs, or abdomen. For Item 6 – Have you gained more than 2 pounds during the past 24 h or more than 5 pounds during the past 72 h, there was minimal variability in responses, with 95% of participants reporting no weight gain. Overall, the study sample demonstrated predominantly asymptomatic behaviour with an adequate perception of their symptoms.

The average HFaST score was 8.43 ± 5.13, with a minimum score of 2 and a maximum of 19.

Concerning the overall score of the KCCQ-23, a mean of 63.83 ± 20.29 was obtained and the mean KCCQ-23 symptom frequency score, encompassing items 3, 5, 7, and 9, was 64.79 ± 25.32. By grouping the scores into four categories to assess health-related QoL: first category (0 to 24), second category (25 to 49), third category (50 to 74) and fourth category (75 to 100), it was found that 2 (3,33%) participants had a very poor to poor QoL, followed by 15 (25%) with poor to fair, 23 (38,33%) with fair to good and 20 (33,33%) with good to excellent. Another noteworthy result was the social limitation score from item 15 of the KCCQ-23 with a mean of 61.67 ± 25.20, suggesting relatively low social limitation in this population, as higher scores reflect better quality of life and fewer restrictions in social activities.

A statistically significant inverse correlation was identified between the social limitation score and the total HFaST score (r_s_​ = −0.615, *p* < 0.001). Patients with lower social limitation scores, representing greater social limitations, tended to have higher HFaST scores, reflecting a more severe symptom burden.

The average time to fill in the HFaST tool was 2.88 ± 1.08 min, and the KCCQ-23 was 4.97 ± 1.08 min.

**Table 3:** HFaST item-level response distributions

HFaST Score	0 – Did not experience in the past 24 h (Não senti nas últimas 24 horas)	1 – Much better than usual (Muito melhor do que o habitual)	2 – Slightly better than usual (Ligeiramente melhor do que o habitual)	3 – About the same as usual (Mais ou menos o mesmo que o habitual)	4 – Slightly worse than usual (Ligeiramente pior do que o habitual)	5 – Much worse than usual (Muito pior do que o habitual)
**HFaST 1** – Fatigue or low energy level when performing everyday activities (Fadiga ou pouca energia ao realizar atividades diárias)	9 (15.0)	5 (8.3)	13 (21.7)	23 (38.3)	10 (16,7)	0 (0)
**HFaST 2** – Shortness of breath when performing everyday activities (Falta de ar quando realiza as atividades diárias)	31 (51.7)	3 (5.0)	7 (11.7)	12 (20.0)	7 (11.7)	0 (0)
**HFaST 3** – Shortness of breath at rest (Falta de ar em repouso)	49 (81.7)	0 (0)	2 (3.3)	7 (11.7)	2 (3.3)	0 (0)
**HFaST 4** – Shortness of breath while lying down or reclining (for example, needing to add pillows or move to a recliner to sleep) (Falta de ar quando deitado ou reclinado, por exemplo, necessidade de adicionar almofadas ou de se mudar para um cadeirão reclinável para dormir)	33 (55.0)	2 (3.3)	4 (6.7)	15 (25.0)	5 (8.3)	1 (1.7)
**HFaST 5** – Swelling of feet, ankles, legs, or abdomen; shoes or waistband feeling tight (Inchaço dos pés, tornozelos, pernas ou abdómen; sensação de sapatos ou cinto apertados)	42 (70.0)	3 (5.0)	3 (5.0)	4 (6.7)	7 (11.7)	1 (1.7)
**HFaST 6** – Have you gained more than 2 pounds during the past 24 h or more than 5 pounds during the past 72 h? (Ganhou mais de 1 kg durante as últimas 24 horas ou mais de 2 kg durante as últimas 72 horas? )	Yes = 3 (5)	No = 57 (95)				

### Reliability and item homogeneity

A Cronbach’s Alpha (α) of 0.724 was obtained, considering it acceptable (α > 0.6).

The results of the inter-item correlation analysis are shown in Table 4. There were moderate correlations between items 1 and 2 (*r* = 0.511), 1 and 3 (*r* = 0.374), 1 and 4 (*r* = 0.390), 2 and 5 (*r* = 0.371), 3 and 4 (*r* = 0.485), and 4 and 5 (*r* = 0.286). Strong correlations were observed between items 2 and 3 (*r* = 0.670) and items 2 and 4 (*r* = 0.683). Inter-item correlations associated with item 6 are notably weaker, compared to the correlations observed among HFaST items 1 through 5. The different response format of item 6 compared to the other items on the HFaST may explain the negative and weak correlations observed for this item. Nevertheless, the inter-item correlations obtained are consistent with the results presented in the original version of the scale [20].

**Table 4:** Inter-item correlations

HFaST Items	Item 1	Item 2	Item 3	Item 4	Item 5	Item 6
**HFaST 1** – Fatigue or low energy level when performing everyday activities (Fadiga ou pouca energia ao realizar atividades diárias)	1					
**HFaST 2** – Shortness of breath when performing everyday activities (Falta de ar quando realiza as atividades diárias)	0.511	1				
**HFaST 3** – Shortness of breath at rest (Falta de ar em repouso)	0.374	0.670	1			
**HFaST 4** – Shortness of breath while lying down or reclining (for example, needing to add pillows or move to a recliner to sleep) (Falta de ar quando deitado ou reclinado, por exemplo, necessidade de adicionar almofadas ou de se mudar para um cadeirão reclinável para dormir)	0.390	0.683	0.485	1		
**HFaST 5** – Swelling of feet, ankles, legs, or abdomen; shoes or waistband feeling tight (Inchaço dos pés, tornozelos, pernas ou abdómen; sensação de sapatos ou cinto apertados)	0.206	0.371	0.076	0.286	1	
**HFaST 6** – Have you gained more than 2 pounds during the past 24 h or more than 5 pounds during the past 72 h? (Ganhou mais de 1 kg durante as últimas 24 horas ou mais de 2 kg durante as últimas 72 horas? )	-0.180	-0.296	-0.151	-0.241	-0.467	1

### Concurrent validity

The Shapiro-Wilk normality test was performed, revealing that the mean scores obtained from the HFaST tool (W = 0.910, *p* < 0.001) did not follow a normal distribution pattern.

The Spearman’s correlation was calculated to assess the concurrent validity between the two questionnaires, as shown in Table 5.

A lower score on each item of the HFaST tool is expected to align with higher scores on the item with equal correspondence on the KCCQ-23 questionnaire. HFaST Item 1 shares an identical evaluation of fatigue with KCCQ-23 Item 5; HFaST Items 2 and 3 are related to the assessment of shortness of breath during activity or at rest, specifically, and correlates with KCCQ-23 Item 7; HFaST Item 4 assesses shortness of breath while lying down and correlates with KCCQ-23 Item 9 and HFaST Item 5 is associated with KCCQ-23 Item 3 to evaluate swelling.

HFaST Item 6 characterised by a dichotomous response, did not demonstrate a correlation with any item in the KCCQ-23 questionnaire.

The moderate to strong correlations found show a negative relationship, justifying not only the opposite behaviour of the two variables under study but also enhancing the statistical significance of our findings.

**Table 5:** Concurrent validity through Spearman’s correlation between HFaST items and KCCQ-23 items

	Spearman’s Rho	*p*-value (2 – tailed)
HFaST item 1 – KCCQ-23 item 5	-0.650**	< 0.001
HFaST item 2 – KCCQ-23 item 7	-0.840**	< 0.001
HFaST item 3 – KCCQ-23 item 7	-0.623**	< 0.001
HFaST item 4 – KCCQ-23 item 9	-0.889**	< 0.001
HFaST item 5 – KCCQ-23 item 3	-0.845**	< 0.001

** Correlation is significant at the 0.01 level (2-tailed)

### Association between hospitalisations and the Portuguese version of the HFaST tool

As hypothesised, hospitalisations due to HF decompensation are related to a higher score on the Portuguese version of the HFaST tool.

**Fig. 3:**
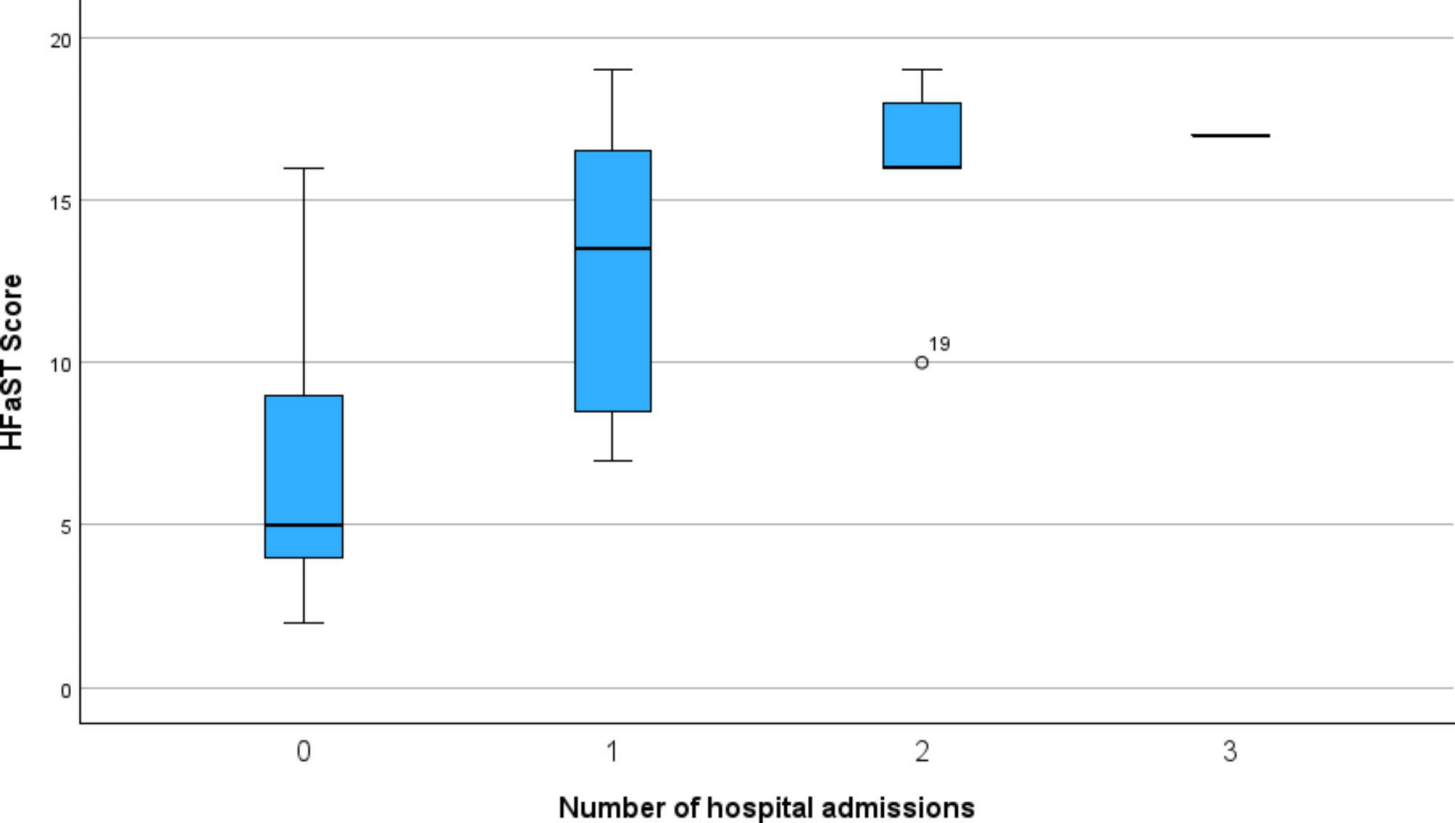
Boxplot showing the distribution of HFaST scores based on the number of hospital admissions

The median HFaST score was considerably higher in patients admitted to the hospital (16; 95% CI: 10–18) compared to non-admitted patients (5; 95% CI: 5–8). This relationship is illustrated in Fig. 3, which shows the distribution of HFaST scores across different numbers of hospital admissions.

Patients with higher scores on the HFaST tended to be more likely to be admitted to the hospital (Mann-Whitney U = 64.500; z = − 4.523; *p* < 0.001).

**Fig. 4:**
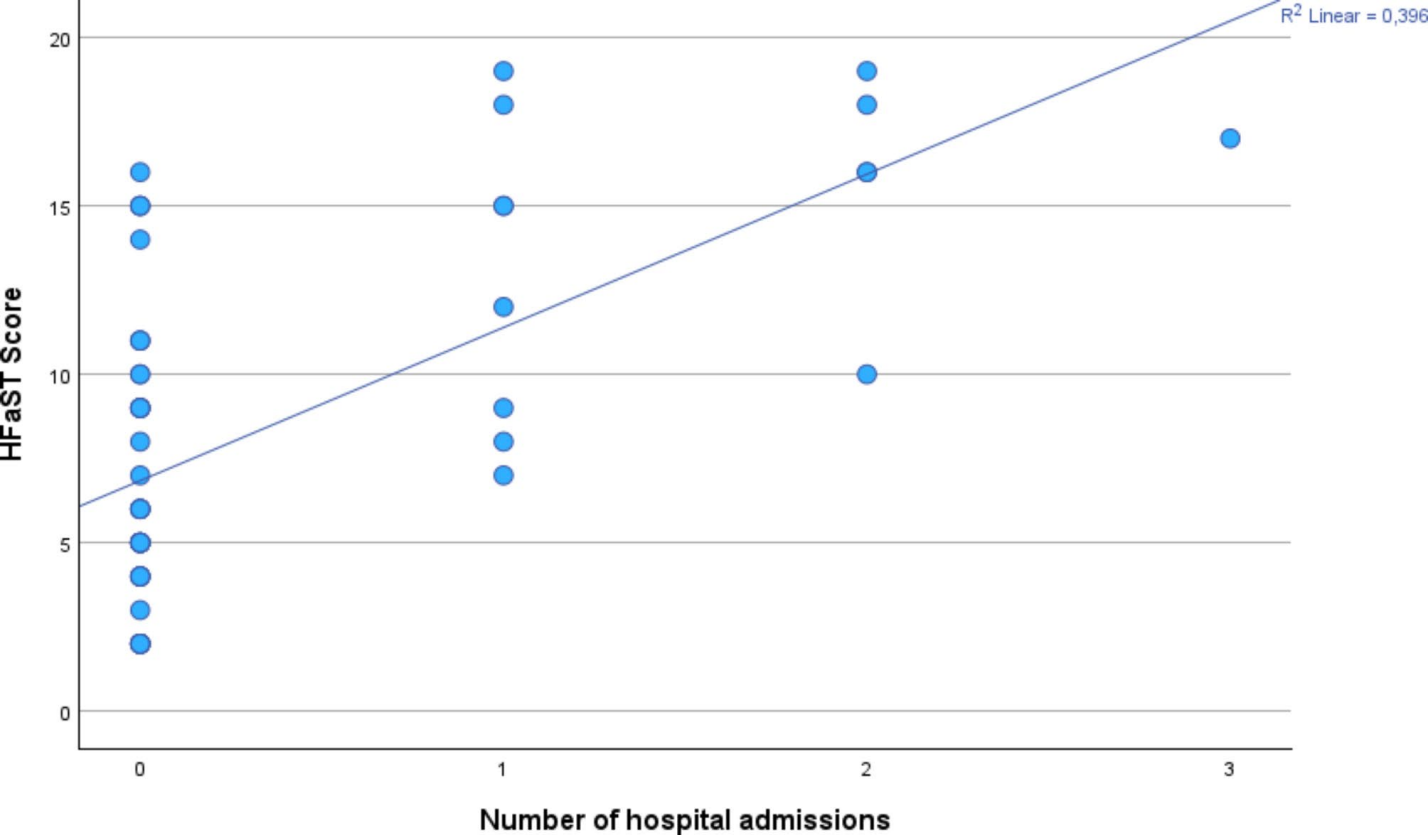
Scatter plot showing the relationship between the number of hospital admissions and HFaST scores. A positive linear association is observed, with higher HFaST scores corresponding to a greater number of hospital admissions. The linear regression model shows an *R²* of 0.396, indicating that about 39.6% of the variability in HFaST scores is explained by the number of hospital admissions

Furthermore, a significant and positive association was observed between the number of hospital admissions and the total HFaST score, as indicated by Spearman’s correlation coefficient of 0.599 (*p* < 0.001). This finding suggests that as the HFaST score increases, the likelihood of hospital admission also increases. This positive linear association is further depicted in Fig. 4.

## Discussion

Health assessment tools, primarily developed in English-speaking countries, require a rigorous process of translation and cross-cultural adaptation, involving an established qualitative and quantitative approach, careful planning and scientific rigour to ensure their validity across diverse populations [21, 22, 24]. On the other hand, specific patient-reported outcomes, such as the KCCQ-23 questionnaire, a golden standard measure for HF, appear to be more accurate and objective, towards a patient-centred approach, being the only questionnaire translated for European Portuguese and validated for the Portuguese population [26, 31, 37].

To fill this gap, this study presents the first translation, cross-cultural adaptation and psychometric validation of the HFaST tool for the Portuguese population, a short and brief questionnaire that allows an assessment of changes in the symptoms presented by HF patients [20].

Our results concerned with experts’ judgement, reported a reasonable agreement on the Portuguese version of the HFaST. However, there was significant disagreement over the size of the Likert scale, with *Fleiss’ k* values for clarity and relevance of 0.548 and 0.570, respectively, which led to the elimination of two items from the Likert scale’s options, reducing their 8-point Likert format to a 6-point format once it could lead to patient confusion and a dubious choice. This decision was further supported by experts’ experience and their qualitative observations, which highlighted that longer Likert scales might not facilitate accurate responses. They suggested that a shorter scale would be less confusing and more user-friendly for patients. Additionally, some studies have shown that cultural differences may make it challenging to interpret the scale or influence responses to the Likert scale. This leads to varying reliability of scale scores due to differences in how respondents interpret and select their choices [38, 39]. This limitation underscores the importance of cultural sensitivity in PROMs’ adaptation and validation. While this study focused on a Portuguese population, the HFaST’s adaptability and reliability in other Portuguese-speaking regions remains untested.

By reducing the number of options on the Likert scale, an adjustment to the scale score was necessary and this was the new Likert scale: *“0 – Did not experience in the past 24 hours/ Não senti nas últimas 24 horas; 1 – Much better than usual/ Muito melhor do que o habitual; 2 – Slightly better than usual/ Ligeiramente melhor do que o habitual; 3 – About the same as usual/ Mais ou menos o mesmo que o habitual; 4 – Slightly worse than usual/ Ligeiramente pior do que o habitual; 5 – Much worse than usual/ Muito pior do que o habitual”* and *“1 – yes/ sim; 2 – no/ não”* for the dichotomous answer on the last item. The new scale score ranges from 1 to 27, with a higher score representing a worse health status and the need for medical care advice.

Our sample characteristics reflect epidemiological trends observed in Portugal, where HF poses a significant and growing health challenge, particularly due to the ageing of the Portuguese population and the life expectancy increase [5, 7]. In Western countries, the prevalence of HF increases with age and ischaemic heart disease and hypertension are predominant factors contributing to HF [5, 7]. The average age of our sample was 63.75 ± 11.77 years. The primary aetiology of HF comprising 43.3% of cases was ischaemic, while 8.3% of participants had hypertensive aetiology. These findings align with the reported trends. LVEF remains HF’s primary diagnostic and prognosis parameter [40]. In this study, 66.6% of the participants exhibited HFrEF, a proportion consistent with findings from similar studies [41].

Therefore, this study revealed that most participants have an adequate perception of symptoms and that lower scores on the HFaST corresponded to a non-symptomatic patient profile, clinically stable and with better health-related quality of life, aligned with the mean KCCQ-23 overall score of 63.83 ± 20.2, indicating a fair to good QoL among most participants in this study. Moreover, the inverse correlation between the social limitation score and the total HFaST score suggests that patients with higher symptom severity tended to experience greater social limitations, highlighting the complex interplay between physical symptoms and psychosocial participation. Previous studies have shown that depression has been associated with a higher risk of adverse outcomes, including rehospitalisation and mortality [42]. Additionally, depressed patients with greater social limitations, tend to report a higher burden of cardiac symptoms compared to those without depression [43].

The HFaST tool was completed in a considerably faster time, 2.09 min less than the KCCQ-23 questionnaire. This demonstrates its practicality as a quick and easy-to-use instrument, making it particularly suitable for clinical context, especially beneficial during follow-up consultations or periodic evaluations, and providing a quick and reliable method for assessing patients’ symptoms.

The Portuguese version of the HFaST demonstrated relevant reliability with an α of 0.724, for a recently developed scale with few items, and exhibited moderate to strong inter-item correlations consistent with the original version [20, 23, 34]. However, weak inter-item correlations were observed for item 6, which can be attributed to its dichotomous response format, differing from the Likert scale used in the other items of the HFaST. Moreover, within our sample, responses to item 6 showed minimal variability, with 95% of participants reporting no weight gain. This lack of variability may reduce the potential for meaningful statistical associations with other items. Despite these findings, the clinical relevance of monitoring subtle weight changes as early indicators of HF decompensation justifies the retention of this item in the scale. Future versions of the HFaST could refine the design of Item 6 to better integrate with the overall structure while maintaining its diagnostic utility.

This study highlighted the critical role of symptom management in patients’ lives, as evidenced by the concurrent validity through correlating KCCQ-23 items with HFaST items [20, 28]. Lower scores on HFaST items corresponded to higher scores on the respective KCCQ-23 items. For instance, HFaST Item 1 and Item 3 demonstrated moderate correlations with their corresponding KCCQ-23 items, with Spearman’s Rho of -0.650 and − 0.623, respectively, suggesting that while the HFaST focuses on capturing specific and actionable clinical indicators, the KCCQ-23 contextualises these symptoms within a broader framework of health-related QoL. This distinction emphasises how the tools complement each other, offering unique but interconnected perspectives on symptom assessment and patient management. Stronger correlations were observed for the remaining HFaST items, such as Item 2, Item 4 and Item 5, which aligned closely with their corresponding KCCQ-23 items, reinforcing the validity of the HFaST in capturing critical clinical markers relevant to HF management. Together, these findings emphasise the statistical significance of our outcomes in the validation of the Portuguese version of the HFaST.

Moreover, symptom awareness plays a crucial role throughout the entire process of managing symptoms [4]. Our results showed that hospital admissions due to HF decompensation correlate with higher scores on the Portuguese version of the HFaST tool. This finding highlights the potential of the HFaST as a practical instrument capable of predicting the likelihood of hospitalisation risk, allowing clinicians and healthcare managers to act earlier in tailored interventions for HF patients.

Nevertheless, the European Society of Cardiology pursues the use of alternative resources more cost-efficiently by managing patients in day-care facilities or outpatient clinics as the hospitals seek to decrease patients’ admissions [26, 44].

Additionally, incorporating the advantages of how HF patients perceive and adopt specific self-monitoring symptom tools, along with their positive effect on reducing hospital admissions and improving health outcomes and quality of care, underlines the growing interest and trend in mHealth technologies, has as a potential solution for symptom self-management [16, 45, 46]. mHealth applications have the potential to enhance HF self-care by providing tools for symptom monitoring and management while improving interactions with healthcare professionals [47].

Our results add to the current literature the positive and high impact of an easy, practical and simple-to-use symptom monitoring and self-management tool, the HFaST, translated, cross-culturally adapted and validated for the Portuguese population, which is expected not only to contribute to better efficiency in managing HF and, consequently, improving patients’ QoL, as also facilitate communication between patient and the medical team.

The key strength of this study is that it might contribute to the use of the Portuguese version of the HFaST tool, first as part of a model for predicting risk and managing hospital admissions and secondly, as part of the health systems transformation, with the engagement of patients in self-management of their chronic diseases with the use of mHealth applications.

Although this study has notable strengths, it is crucial to recognise some limitations. First, to support the validity and reliability of the HFaST, a larger sample is required to confirm these psychometric properties and evaluate the tool in more diverse populations. The study population focused only on outpatients, excluding hospitalised patients who may present more severe symptoms and different patterns of disease progression. Future research should aim to include larger, multicentre cohorts encompassing both outpatients and hospitalised HF patients, to ensure a more heterogeneous population and broader applicability of the findings.

Another limitation is the focus on NYHA I or II patients, limiting its applicability to more symptomatic populations. Future research should explore the HFaST’s performance in advanced NYHA stages and consider factors like LVEF to enhance its applicability and address hospitalisation risks.

Future studies should focus on transcultural validation of the HFaST in other Portuguese-speaking regions to assess variability in symptom interpretation and response patterns. This will enhance the tool’s applicability and clinical utility across diverse Lusophone populations.

A comparative analysis between different local health units could provide insights into regional variations in HF management and outcomes. Additionally, comparing the HFaST with other symptom trackers, such as the AHA tool, could highlight their strengths and weaknesses, offering insight into their impacts on symptom management and patient outcomes. Finally, a cost-effectiveness analysis would be beneficial to understand the impact of a symptom self-management tool on potentially reducing hospitalisation admissions rates.

## Conclusions

The Portuguese version of the HFaST is an important tool for quick and straightforward self-assessment of symptoms in HF patients and has demonstrated reliable psychometric validity, showing strong correlations with the KCCQ-23 questionnaire and a significant predictive association with hospitalisations. This emphasises the importance of patient self-monitoring, making it a powerful tool for HF patients in the Portuguese context, bringing clear benefits for better disease management, improving QoL and reducing hospital admissions.

## Data Availability

The data underlying this article can be acquired from the corresponding author upon a reasonable request.

## Abbreviations

α: Cronbach’s Alpha
AHA: American Heart Association
HF: Heart Failure
HFaST: Heart Failure Symptom Tracker
HFrEF: Heart Failure with Reduced Ejection Fraction
HFpEF: Heart Failure with Preserved Ejection Fraction
I-CVI: Item Content Validity Index
KCCQ-23: Kansas City Cardiomyopathy Questionnaire
LVEF: Left Ventricular Ejection Fraction
MLHF: Minnesota Living with Heart Failure Questionnaire
mHealth: Mobile Health
NYHA: Hew York Heart Association
PROMs: Patient-Reported Outcome Measures
QoL: Quality of Life
S-CVI: Scale Content Validity Index
